# Transfusion in Anæmia

**Published:** 1880-09

**Authors:** 


*Collaborators:*

DR. L. W. CASE, DR. R. TILLEY,
DR. BYRON W. GRIFFIN, DR. W. L. DORLAND.

### TRANSFUSION IN ANÆMIA.

Dr. DeCailhol, in *Ohio Medical Recorder*, gives a case of transfusion in extreme anæmia after the manner of Dr. Moncoy, the French specialist, using nondefibrinated blood by direct transfusion from the basilic vein of the right arm of the husband of the patient, a strong, well developed man, absolutely free from constitutional taint, into the patient's right arm through the median basilic vein. Two ounces of blood only were transfused, but the pulse instantly rose and the patient felt warmer and more comfortable. Rest and milk diet prescribed, with two ounces of warm defibrinated beef blood, and two ounces rich soup made of beef and pork pancreas every four hours by enema, the stomach tolerating nothing but the milk. In twenty days the operation was repeated and an ounce and a half transfused; treatment continued. The stomach then able to tolerate a small quantity of beef tea to which was added pepsine. A third and last operation (performed with difficulty on account of the patient's marked improvement), occurred in eighteen days. Two days subsequently the stomach tolerated chicken, and she was able, gradually, to use more solid food. In forty days after the last transfusion, the patient started for Europe and returned in two months with plump and rosy cheeks and every appearance of good health.

The doctor remarks in summarizing the case that no iron was given after the first experiment which the stomach refused to tolerate, and he firmly believes the cure due to the four and a half ounces of healthy blood transfused. He has known of many unsuccessful cases of transfusion in the United States, which can be attributed to the neglect of various minutiæ in the operation, such as the use of improper or imperfect instruments; the use of defibrinated blood; woman's blood; milk; or after too much manipulation of the vein, all just so many causes of failure. Dr. DeC. has never met with failure in following strictly the rules laid down by the French specialist in his monogram on the subject (which, by the way, Dr. DeC. intends translating for the benefit of the American profession), the most imperative of which are: to use none other than the blood from a perfectly sound man between twenty and forty years of age. The blood must not be defibrinated; it must be transfused slowly, not in a large stream lest the heart be taken by surprise. He uses nondefibrinated blood because the assimilation is to be made at once, without alteration in the blood. He thinks blood loses by the removal of its fibrin the best part of its constituent, and is thereby rendered unfit and even dangerous to the circulation. To the timid operator who fears coagulation of the blood during transfusion, he says no such thing ever takes place if the surgeon is careful and expert and has everything ready at hand; is well assisted and uses a suitable instrument properly handled and not over-heated, (though he does not give the one he uses), as the blood clots sooner by heat than cold. The surgeon must bear in mind that he has from three to four minutes before him to transfuse before clotting will take place, and this is ample time for the transmission of two ounces or more.

As to the defibrination of the animal blood used by enema, it is thus used because it will not keep twenty-four hours; also it is intended for food and does not go immediately into circulation.

